# Entropy Generation during Head-On Interaction of Premixed Flames with Inert Walls within Turbulent Boundary Layers

**DOI:** 10.3390/e24040463

**Published:** 2022-03-27

**Authors:** Sanjeev Kr. Ghai, Umair Ahmed, Nilanjan Chakraborty, Markus Klein

**Affiliations:** 1 School of Engineering, Newcastle University, Newcastle-upon-Tyne NE1 7RU, UK; sanjeev.ghai@newcastle.ac.uk (S.K.G.); umair.ahmed@newcastle.ac.uk (U.A.); nilanjan.chakraborty@newcastle.ac.uk (N.C.); 2 Department of Aerospace Engineering, University of the Bundeswehr Munich, 85577 Neubiberg, Germany

**Keywords:** entropy generation, head-on interaction, turbulent premixed flame, viscous action, chemical reaction, thermal diffusion, molecular mixing, second-law efficiency

## Abstract

The statistical behaviours of different entropy generation mechanisms in the head-on interaction of turbulent premixed flames with a chemically inert wall within turbulent boundary layers have been analysed using Direct Numerical Simulation data. The entropy generation characteristics in the case of head-on premixed flame interaction with an isothermal wall is compared to that for an adiabatic wall. It has been found that entropy generation due to chemical reaction, thermal diffusion and molecular mixing remain comparable when the flame is away from the wall for both wall boundary conditions. However, the wall boundary condition affects the entropy generation during flame-wall interaction. In the case of isothermal wall, the entropy generation due to chemical reaction vanishes because of flame quenching and the entropy generation due to thermal diffusion becomes the leading entropy generator at the wall. By contrast, the entropy generation due to thermal diffusion and molecular mixing decrease at the adiabatic wall because of the vanishing wall-normal components of the gradients of temperature and species mass/mole fractions. These differences have significant effects on the overall entropy generation rate during flame-wall interaction, which suggest that combustor wall cooling needs to be optimized from the point of view of structural integrity and thermodynamic irreversibility.

## 1. Introduction

Entropy generation is of fundamental importance in energy generation processes because it provides the measure of loss of work potential due to different sources of irreversibility [1]. This aspect plays a key role in determining the efficiency in internal combustion (IC) engines and gas turbines. Industrial combustors are increasingly made smaller in size to increase their energy-density and to make them compatible for hybrid-electrical powertrains. The reduced size of combustors increases the possibility of flame-wall interaction and this could lead to flame quenching due to wall heat loss caused by higher surface area to volume ratio of the combustion chamber. Therefore, it is necessary to analyse the statistics of entropy generation during flame-wall interaction (FWI) to identify the evolutions of irreversibility as the flame surface interacts with the wall in order to optimise the performance of new generation combustors. In particular, entropy generation in the case of FWI is yet to be analysed in detail. This motivates the current analysis which focuses on the statistical behaviours of the different entropy generation mechanisms and their relative contributions to the overall thermodynamic irreversibility in head-on interaction of premixed flames with chemically inert walls within turbulent boundary layers, as FWI in most industrial flows takes place within turbulent boundary layers.

The entropy generation due to viscous action and thermal conduction during heat transfer in non-reacting boundary layers was investigated by Arpaci and Selamat [2]. The contributions of entropy generation due to thermal diffusion and molecular mixing have been analysed by San et al. [3] and Poulikakos and Johnson [4] for non-reacting flows. In reacting flows, the entropy generations due to viscous action, molecular mixing and thermal diffusion are simultaneously at play but there is an additional contribution due to chemical reaction rate. Although entropy generation mechanisms for turbulent FWI are yet to be analysed in detail, several previous analyses [5,6,7,8,9,10,11,12,13,14,15,16,17,18,19] focused on entropy generation and irreversibility analysis for reacting flows away from walls. Dunbar and Lior [5] conducted system level analysis for oxidation of methane and hydrogen and revealed that the entropy generations due to molecular mixing and thermal diffusion play key roles in reducing the second-law efficiency. Teng et al. [6] demonstrated that mixing and differential diffusion can augment entropy generation rate in multicomponent reactive systems. The entropy generation in spray combustion was analysed by Puri [7] and the optimum transfer number was identified for the minimisation of the entropy generation. Hiwase et al. [8] provided the theoretical background for entropy generation in droplet-laden combustion as a function of Damköhler number and initial droplet temperature. Exergy analysis for spray combustion was also analysed by Som and Datta [9] for different operating conditions in terms of pressures, temperatures, swirl number and cone-angle. Arpaci and Selamat [10] used analytical tools to express entropy generation rate in terms of quenching distance for a flat laminar premixed flame anchored to a porous-plug flameholder. Nishida et al. [11] used detailed chemistry laminar methane-air and hydrogen-air premixed flame simulations to analyse the entropy generation rates and reported that equivalence ratio significantly affects the entropy generation mechanisms in premixed flames. The entropy generation mechanisms in a laminar diffusion flame were analysed by Datta [12], whereas the effects of hydrogen-blending on entropy generation rate in laminar methane-air premixed flames have been analysed by Briones et al. [13]. Som et al. [14] analysed exergy balance based on Reynolds Averaged Navier-Stokes (RANS) simulations of turbulent flame impingement heat transfer. O’Kongo and Bellan [15] analysed entropy production in a supercritical n-heptane/N_2_ turbulent mixing layer using high-fidelity three-dimensional Large Eddy Simulations (LES). Safari et al. [16] analysed the implications of local entropy generation rates on combustion modelling of non-premixed flames in the context of LES. The available literature on thermodynamic exergy analysis and exergy balance in combustion systems until the first half of the decade of 2000 was reviewed by Som and Datta [17]. Subsequently, the advancements in high-performance computing have enabled exergy analysis of turbulent combustion processes based on LES and Direct Numerical Simulations (DNS). Farran and Chakraborty [18] analysed entropy generation characteristics in statistically planar turbulent premixed flames for different turbulence intensities, heat release parameters and characteristic Lewis numbers using three-dimensional DNS data. They demonstrated that the regime of combustion does not have a significant influence on the augmentation of entropy generation rate in comparison to the unstretched laminar flames. Chakraborty [19] utilised the DNS data to propose models for the mean contributions of entropy generation due to viscous action, thermal diffusion, molecular mixing and chemical reaction in terms of dissipation rate of kinetic energy and scalar dissipation rate in the framework of RANS. All of these aforementioned analyses for entropy production in combustion processes were carried out in configurations without the influence of walls. Recent LES analyses [20,21,22] for non-reacting wall-bounded flows with heat transfer revealed that the presence of a wall and the associated boundary layer have significant influence on entropy generation rates. Therefore, it can be expected that the presence of a wall will have a significant influence on the entropy generation rate in premixed flames due to three-way interactions between wall, fluid flow and chemical processes within the boundary layer, but insufficient information is available in the literature for exergy analysis for turbulent premixed FWI. To address this gap in the existing literature, the current analysis uses three-dimensional DNS data of statistically planar turbulent premixed flames propagating across the turbulent boundary layer towards a chemically inert wall to analyse the entropy generation mechanisms during head-on interactions of the flame with the wall. These entropy generation mechanisms have been analysed for both isothermal and adiabatic boundary conditions at the wall to understand the influence of thermal boundary conditions on the entropy generation and also on the second-law efficiency. The main objectives of this analysis are: (1) to demonstrate the evolution of the relative contributions of different entropy generation contributions at different stages of head-on interaction of turbulent premixed flames with the wall, (2) to demonstrate the impact of thermal boundary condition on the entropy generation rate characteristics during head-on interaction of turbulent premixed flames with the wall and (3) to provide physical explanations for the observations made in the context of objectives 1 and 2.

The rest of the paper will be organised as follows. The mathematical framework and the numerical implementation pertaining to the current analysis are presented in Section 2 and Section 3, respectively. This will be followed by the presentation of the results and their discussion in Section 4. The main findings are summarised and conclusions are drawn in the final section of this paper.

## 2. Mathematical Framework

The volumetric rate of exergy destruction is given as [1,16,18]:

(1) $${\dot{E}}_{d}=T_{0}S_{gen}^{\prime \prime \prime }$$

Here, $T_{0}$ is the unburned gas temperature, which is taken to be the dead state for the purpose of this analysis and $S_{gen}^{\prime \prime \prime }$ is the volumetric entropy generation rate which can be obtained from the transport equation of specific entropy s. The transport equation of specific entropy s takes the following form [16,18]:

(2) $$T\rho \frac{Ds}{Dt}=\rho \frac{De}{Dt}+p\rho \frac{D\vartheta }{Dt}-\overset{N}{\underset{\alpha =1}{\sum }}\mu_{\alpha }\rho \frac{DY_{\alpha }}{Dt}$$

where $T,e,\rho ,p,\vartheta ,\mu_{\alpha }$ and $Y_{\alpha }$ are the temperature, specific internal energy, density, thermodynamic pressure, specific volume, specific chemical potential of species α (i.e., per unit mass and not per unit mole) and mass fraction of species α, respectively. The specific chemical potential $\mu_{\alpha }$ is expressed as [16,18,23]:

(3) $$\mu_{\alpha }=h_{\alpha }-Ts_{\alpha }^{o}$$

Here, $h_{\alpha }=h_{\alpha }^{o}+\int_{T_{ref}}^{T}c_{p\alpha }dT$ is the specific enthalpy of species α, $c_{p\alpha }$ is the specific heat of species α, $T_{ref}$ is the reference temperature and $s_{\alpha }^{o}$ is the specific partial entropy which is expressed as [16,18,23]:

(4) $$s_{\alpha }^{o}=\frac{1}{M_{\alpha }}{\left(\frac{\partial S}{\partial n_{\alpha }}\right)}_{p,T,n_{\beta }\left(\beta \neq \alpha \right)}$$

Here, $M_{\alpha }$ is the molecular weight and $n_{\alpha }$ is the number of moles of species α and $S=\overset{N}{\underset{\alpha =1}{\sum }}m_{\alpha }s_{\alpha }$ is the extensive entropy. For the purpose of this analysis, all the species are considered to be ideal gases, which leads to [16,18,23]:

(5) $$s_{\alpha }^{o}=s_{\alpha }-R_{\alpha }\ln X_{\alpha }$$

In Equation (5), $s_{\alpha }$, $X_{\alpha }$ and $R_{\alpha }=R^{o}/M_{\alpha }$ are the specific entropy, mole fraction and gas constant for species α, respectively and $R^{o}$ is the universal gas constant. Using Equations (2)–(5), it is possible to obtain the transport equation of specific entropy [16,18,23]:

(6) $$\frac{\partial \left(\rho s\right)}{\partial t}+\frac{\partial \left(\rho u_{j}s\right)}{\partial x_{j}}=\frac{1}{T}\left[\tau_{ij}\frac{\partial u_{i}}{\partial x_{j}}-\frac{\partial q_{i}}{\partial x_{i}}+\overset{n}{\underset{i=1}{\sum }}\mu_{\alpha }\frac{\partial J_{i}^{\alpha }}{\partial x_{i}}-\overset{n}{\underset{i=1}{\sum }}\mu_{\alpha }{\dot{w}}_{\alpha }\right]$$

where $\tau_{ij}$ is the component of viscous stress, $q_{i}$ is the *i*th component of the heat flux and $J_{i}^{\alpha }$ is the diffusion mass flux for species α, which are expressed according to Newton’s law of viscosity, Fourier’s law and Fick’s law respectively in the following manner:

(7) $$\tau_{ij}=\mu \left(\frac{\partial u_{i}}{\partial x_{j}}+\frac{\partial u_{j}}{\partial x_{i}}\right)-\frac{2\mu }{3}\delta_{ij}\left(\frac{\partial u_{k}}{\partial x_{k}}\right);\;q_{i}=-\lambda \frac{\partial T}{\partial x_{i}}+\sum_{i=1}^{n}h_{\alpha }J_{i}^{\alpha };\;J_{i}^{\alpha }=-\rho D_{\alpha }\frac{\partial Y_{\alpha }}{\partial x_{i}}$$

Here, μ is the dynamic viscosity, λ is the thermal conductivity, $D_{\alpha }$ is the mass diffusivity of species α and its mass fraction is denoted by $Y_{\alpha }$. Using Equation (7) in Equation (6) provides [16,18,23]:

(8) $$\begin{array}{cc} \frac{\partial \left(\rho s\right)}{\partial t}+\frac{\partial \left(\rho u_{j}s\right)}{\partial x_{j}} & =\frac{1}{T}[\tau_{ij}\frac{\partial u_{i}}{\partial x_{j}}-\frac{\left(q_{i}-\sum_{\alpha =1}^{n}h_{\alpha }J_{i}^{\alpha }\right)}{T}\frac{\partial T}{\partial x_{i}}+\sum_{\alpha =1}^{n}J_{i}^{\alpha }\;\left(\frac{\partial \mu_{\alpha }}{\partial x_{i}}+s_{\alpha }^{o}\frac{\partial T}{\partial x_{i}}\right)\\ & -\sum_{i=1}^{n}\mu_{\alpha }{\dot{w}}_{\alpha }]-\frac{\partial }{\partial x_{i}}\left[\frac{\left(q_{i}-\sum_{\alpha =1}^{n}h_{\alpha }J_{i}^{\alpha }\right)}{T}+\sum_{\alpha =1}^{n}s_{\alpha }^{o}J_{i}^{\alpha }\right] \end{array}$$

Moreover, the third term on the right-hand side of Equation (8) can be expressed as [16,18,23]:

(9) $$\overset{n}{\underset{\alpha =1}{\sum }}J_{i}^{\alpha }\;\left(\frac{\partial \mu_{\alpha }}{\partial x_{i}}+s_{\alpha }^{o}\frac{\partial T}{\partial x_{i}}\right)=\overset{n}{\underset{\alpha =1}{\sum }}\frac{J_{i}^{\alpha }}{T}\left(c_{p\alpha }\frac{\partial T}{\partial x_{i}}-T\frac{\partial s_{\alpha }^{o}}{\partial x_{i}}\right)\;$$

Using Equation (5), $\partial s_{\alpha }^{o}/\partial x_{i}$ can be estimated as [16,18,23]:

(10) $$\frac{\partial s_{\alpha }^{o}}{\partial x_{i}}=\frac{c_{p\alpha }}{T}\frac{\partial T}{\partial x_{i}}-\frac{R_{\alpha }}{X_{\alpha }}\frac{\partial X_{\alpha }}{\partial x_{i}}-\underset{=0}{\underbrace{\frac{R_{\alpha }}{p}\frac{\partial p}{\partial x_{i}}}}$$

The last term on the right-had side of Equation (10) is ignored for low Mach number flows [16,18,23]. Using Equation (10) in Equation (9) yields [16,18,23]:

(11) $$\overset{n}{\underset{\alpha =1}{\sum }}J_{i}^{\alpha }\;\left(\frac{\partial \mu_{\alpha }}{\partial x_{i}}+s_{\alpha }^{o}\frac{\partial T}{\partial x_{i}}\right)=\overset{n}{\underset{\alpha =1}{\sum }}\;J_{i}^{\alpha }\frac{R_{\alpha }}{X_{\alpha }}\frac{\partial X_{\alpha }}{\partial x_{i}}$$

Thus, the last term on the right-hand side of Equation (8) takes the following form [16,18,23]:

(12) $$-\frac{\partial }{\partial x_{i}}\left[\frac{\left(q_{i}-\sum_{\alpha =1}^{n}h_{\alpha }J_{i}^{\alpha }\right)}{T}+\overset{n}{\underset{\alpha =1}{\sum }}s_{\alpha }^{o}J_{i}^{\alpha }\right]=\frac{\partial }{\partial x_{i}}\left[\frac{\lambda }{T}\frac{\partial T}{\partial x_{i}}+\overset{N}{\underset{i=1}{\sum }}\rho s_{\alpha }^{o}D_{\alpha }\frac{\partial Y_{\alpha }}{\partial x_{i}}\right]$$

Here, all the specific heats and mass diffusivities are considered to be identical (i.e., $c_{p\alpha }=c_{p}$ and $D_{\alpha }=D$) for all species, which enables one to write:

(13) $$\frac{\partial }{\partial x_{i}}\left[\frac{\lambda }{T}\frac{\partial T}{\partial x_{i}}+\overset{N}{\underset{i=1}{\sum }}\rho s_{\alpha }^{o}D_{\alpha }\frac{\partial Y_{\alpha }}{\partial x_{i}}\right]=\frac{\partial }{\partial x_{i}}\left(\rho D\frac{\partial s}{\partial x_{i}}\right)+\frac{\partial }{\partial x_{i}}\left[\frac{\lambda }{T}\left(1-\frac{1}{Le}\right)\frac{\partial T}{\partial x_{i}}\right]$$

where $Le=\lambda /\rho c_{p}D$ is the Lewis number. Using Equations (11) and (13) in Equation (8) provides the transport equation of entropy in the following form [16,18,23]:

(14) $$\frac{\partial \left(\rho s\right)}{\partial t}+\frac{\partial \left(\rho u_{j}s\right)}{\partial x_{j}}=\frac{\partial }{\partial x_{i}}\left(\rho D\frac{\partial s}{\partial x_{i}}\right)+\frac{\partial }{\partial x_{i}}\left[\frac{\lambda }{T}\left(1-\frac{1}{Le}\right)\frac{\partial T}{\partial x_{i}}\right]+S_{1}+S_{2}+S_{3}+S_{4}$$

where the terms $S_{1},S_{2},S_{3}$ and $S_{4}$ are given by [16,18,23]:

(15) $$S_{1}=\frac{1}{T}\left(\tau_{ij}\frac{\partial u_{i}}{\partial x_{j}}\right);\;S_{2}=\frac{-\sum_{i=1}^{n}\mu_{\alpha }{\dot{w}}_{\alpha }}{T};\;S_{3}=\frac{\lambda }{T^{2}}\frac{\partial T}{\partial x_{i}}\frac{\partial T}{\partial x_{i}};\;S_{4}=\overset{N}{\underset{\alpha =1}{\sum }}\frac{\rho DR_{\alpha }}{X_{\alpha }}\frac{\partial X_{\alpha }}{\partial x_{i}}\frac{\partial Y_{\alpha }}{\partial x_{i}}$$

It is evident from Equation (15) that $S_{1}$ represents entropy generation due to viscous action, whereas $S_{2}$ denotes the entropy generation as a result of chemical reaction. The entropy generation due to thermal diffusion is represented by $S_{3}$ and the entropy generation due to molecular mixing is denoted by $S_{4}$. The terms on the left-hand side of Equation (15) represent transient and advection terms, respectively. The first term on the right-hand side represents the molecular diffusion of entropy, whereas the second term on the right-hand side represents redistribution of entropy due to non-unity Lewis number. However, the volume integral of ∇·[λ/T(1−1/Le)∇T] vanishes according to the divergence theorem and thus, does not contribute to entropy generation. Therefore, the total volumetric entropy generation rate is given by [16,18,23]:

(16) $$S_{gen}^{\prime \prime \prime }=S_{1}+S_{2}+S_{3}+S_{4}$$

For the purpose of the current analysis, the (turbulent) augmentation of an entropy generation mechanism can be quantified as $Q_{T_{i}}=\int_{V_{f}}{\left(S_{i}\right)}_{turb}dV/\left[\int_{V_{f}}{\left(S_{i}\right)}_{lam}dV\right]$ (where i=1,2,3 and 4) [18] and the overall (turbulent) entropy generation enrichment is expressed as: $Q_{T}=\int_{V_{f}}{\left(S_{1}+S_{2}+S_{3}+S_{4}\right)}_{turb}dV/\int_{V_{f}}{\left(S_{1}+S_{2}+S_{3}+S_{4}\right)}_{lam}dV$ with subscripts ‘turb’ and ‘lam’ referring to values under turbulent and laminar conditions and $V_{f}$ is the flame volume. The flame volume $V_{f}$ is defined as the volume given by the 0.001≤c≤0.999 and $0.001\leq \tilde{c}\leq 0.999$ for laminar and turbulent flames, respectively, where $c=\left(Y_{F}-Y_{Fu}\right)/\left(Y_{Fb}-Y_{Fu}\right)$ is the reaction progress variable based on fuel (F) mass fraction $Y_{F}$. The reaction progress variable c rises monotonically from 0 in the unburned gas (values in the unburned gas shown by the subscript *u*) to 1.0 in the fully burned gas (values in the burned gas shown by the subscript *b*) and $\tilde{c}=\bar{\rho c}/\bar{\rho }$ is the Favre-averaged value of reaction progress variable with the overbar (tilde) suggesting a Reynolds (Favre) averaging process.

Finally, the second-law efficiency in this configuration is defined based on irreversibilities in the following manner:

(17) $$\eta_{II}=1-\frac{\int_{V_{f}}T_{0}S_{gen}^{\prime \prime \prime }dV}{\int_{V_{f}}\sum_{i=1}^{n}\left(-a_{\alpha }{\dot{w}}_{\alpha }\right)dV+\int_{A}q_{w}dA}$$

where $a_{\alpha }$ is the specific availability of species α at temperature $T_{0}$ and $q_{w}$ is the wall heat flux magnitude. The statistical behaviours of $S_{i}$,$\;Q_{T_{i}}$ (for i=1, 2, 3 and 4), $Q_{T}$ and $\eta_{II}$ at different stages of head-on interaction of turbulent premixed flames with a chemically inert wall for both isothermal and adiabatic thermal boundary conditions will be discussed in Section 4 of this paper.

## 3. Numerical Implementation

**Figure 1:**
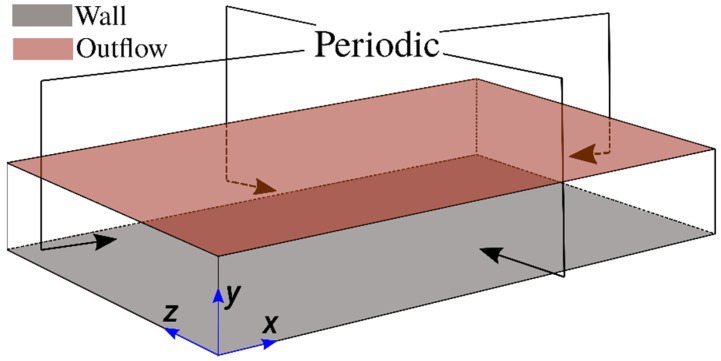
Schematic diagram of the head-on interaction configuration considered here.

The entropy generation characteristics in head-on interaction of turbulent premixed flames with chemically inert walls are analysed based on three-dimensional DNS data. The schematic diagram for the simulation configuration is shown in Figure 1. The simulations have been conducted using a three-dimensional code called SENGA+ [24] and the combustion chemistry is represented by a single-step Arrhenius type chemical reaction (unit mass of Fuel + s unit mass of Oxidiser → (1 + s) unit mass of Products, where s is the stoichiometric oxidiser-fuel mass ratio) for the sake of computational economy. It is worth noting several previous analyses [25,26,27,28,29,30,31] used single-step chemistry in order to analyse turbulent premixed FWI and the same approach has been adopted in this study. The statistics of reactive scalar gradient, wall heat flux magnitude and the flame quenching distance obtained from detailed chemistry simulations have been found to be qualitatively similar even for single-step chemistry, which was discussed in detail elsewhere [32,33] and, thus, will not be repeated here. It is worth noting that the SENGA+ code is well established and used in several previous studies [18,19,29,30,31,32,33] including entropy transport analysis [18,19]. Exemplarily, for a Taylor-Green-Vortex test case, the maximum deviation in enstrophy from SENGA+ simulation with respect to reference data [34,35] is 2.5%, while kinetic energy can be considered to be identical with the aforementioned reference solutions. The parallel performance of SENGA+ has been demonstrated in Refs. [36,37] in the past and, thus, is not repeated here.

For the purpose of this analysis, a stoichiometric methane-air mixture (i.e., s=4.0) under atmospheric conditions is considered. The unburned gas temperature $T_{0}$ is taken to be 730 K, which gives rise to a Zeldovich parameter, $\beta =T_{a}\left(T_{ad}-T_{0}\right)/T_{ad}^{2}$ of 6.0 (where $T_{a},\;T_{ad},\;T_{0}$ are the activation, adiabatic and reactant temperatures, respectively) and a heat release rate parameter of $\tau =(T_{ad}-T_{0})/T_{0}=2.3$. Standard values are taken for the Prandtl number Pr and the ratio of specific heat, γ (i.e., Pr=0.7, γ=1.4). In SENGA+, the spatial derivatives are evaluated using a 10th order finite difference central scheme for the internal grid points, whereas the order of accuracy gradually reduces to second order for the non-periodic boundaries. A third-order Runge-Kutta scheme has been employed for time advancement. The initial flow conditions have been generated by a non-reacting turbulent channel flow solution corresponding to $Re_{\tau }=\rho_{0}u_{\tau ,NR}h/\mu_{0}=110$ where $\rho_{0}$ is the unburned gas density, $\mu_{0}$ is the unburned gas viscosity and h is the channel half height corresponding to the non-reacting fully developed channel flow solution. The computational domain is taken to be 10.69h×1.33h×4h with an equidistant grid resolution of 1920×240×720, which ensures at least 8 grid points within the thermal flame thickness $\delta_{th}=(T_{ad}-T_{0})/\max{\left|\nabla T\right|}_{L}$ for $S_{L}/u_{\tau ,NR}=0.7$ where $S_{L}$, $u_{\tau ,NR}=\sqrt{\left|\tau_{w,NR}\right|/\rho }$ and $\tau_{w,NR}$ are the unstretched laminar burning velocity, friction velocity and wall shear stress for the non-reacting channel flow, respectively. For the channel flow configuration, the longitudinal integral length scale $L_{11}$ remains of the order of h and the root-mean-square turbulent velocity scales with $u_{\tau ,NR}$ [38], which suggest a Damköhler number $Da=L_{11}S_{L}/u^{\prime }\delta_{th}$ of 15.80 and a Karlovitz number $Ka={\left(u^{\prime }/S_{L}\right)}^{3/2}{\left(L_{11}/\delta_{th}\right)}^{-1/2}$) of 0.36. These values are representative of the corrugated flamelets regime combustion [39]. The simulations have been conducted for a Mach number of $Ma=u_{\tau }/a_{0}=3\times 10^{-3}$ where $a_{0}$ is the acoustic speed in the unburned gas.

For these simulations, periodic boundaries are considered for the streamwise (i.e., x-direction) and spanwise (i.e., z-direction) directions and the mean pressure gradient (i.e., $-\partial p/\partial x=\rho u_{\tau ,NR}^{2}/h$ where p is the pressure) has been imposed in the streamwise flow direction, as shown in Figure 1. In the wall-normal direction (i.e., y-direction), a no-slip boundary condition is implemented at y=0, whereas a Dirichlet boundary condition is specified (i.e., $T_{w}=T_{0}$) for the isothermal wall boundary condition. However, a Neumann boundary condition, given by ∂T/∂y=0 , is used for the adiabatic wall boundary condition. A partially non-reflecting boundary is specified at y/h=1.33 according to the Navier-Stokes Characteristic Boundary Conditions (NSCBC) conditions proposed by Yoo and Im [40]. The flow configuration used in the present work is similar to the configuration used in the earlier work of Bruneaux et al. [27,28]. However, in contrast to Bruneaux et al. [27,28], the current simulations account for the variation of density due to temperature change and outflow boundary conditions are implemented to avoid any change in the thermodynamic pressure due to density variation caused by combustion. In the current simulation setup, the solution from the 1-D laminar flame simulation is interpolated initially to the 3-D grid in such a manner that the reaction progress variable c = 0.5 is obtained at y/h≈0.85. The reacting scalar field is initialised in a manner that the reactant side of the flame faces the wall, whereas the product side of the flame faces towards the outflow side of the boundary in the y-direction. The simulations were conducted for 2.0 flow through times based on the maximum streamwise mean velocity, which is equal to $21.30\;u_{\tau ,NR}$. Within the duration of the simulation time, the flame propagates and moves towards the wall and interacts with it. By contrast, the boundary layer does not evolve significantly during the course of the simulation [31].

The Reynolds and Favre averaged quantities involving correlations of Reynolds and Favre fluctuations have been calculated by spatial averaging the quantities of interest in the statistically homogeneous periodic directions (i.e., x−z planes) for a given time instant [30,31].

## 4. Results and Discussion

**Figure 2:**
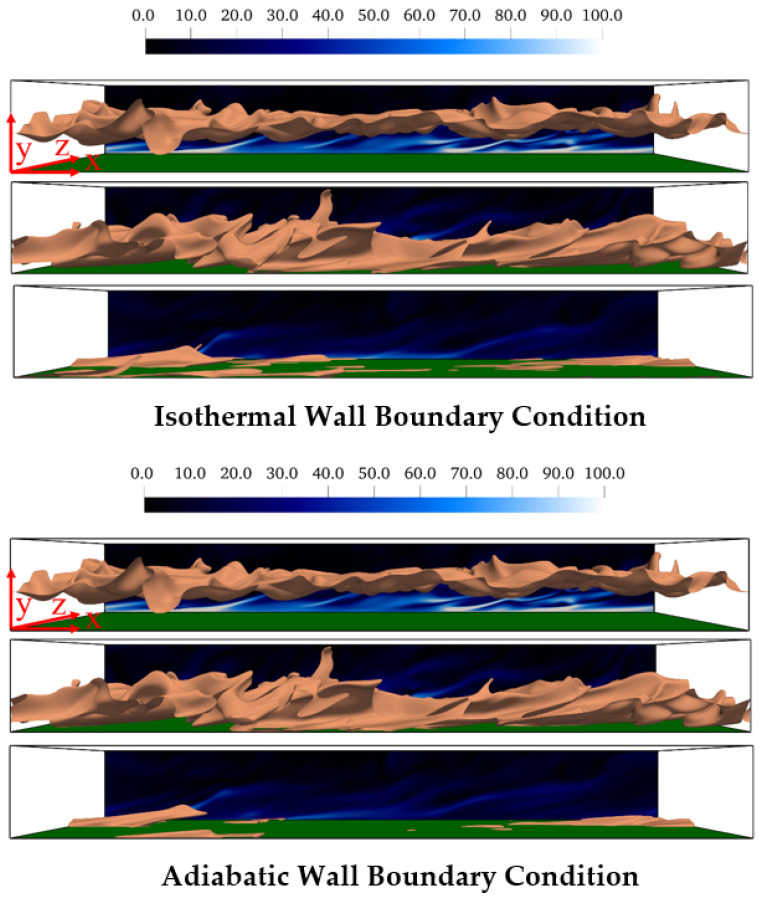
Head-on interaction with isothermal (**top**) and adiabatic (**bottom**) wall boundary conditions at $t/t_{f}=4.20$, 10.50, 14.70 (**top** to **bottom**). The isosurface coloured in peach represents c=0.5. The instantaneous normalised vorticity magnitude $Ω=\sqrt{\omega_{i}\omega_{i}}\times h/u_{\tau ,NR}$ is shown on the x−y plane at z/h=4. The green surface denotes the wall.

The temporal evolutions of head-on wall interaction of turbulent premixed flames propagating across the boundary layer are shown in Figure 2 for both isothermal and adiabatic wall boundary conditions. The reaction progress variable c=0.5 isosurface is shown in red and the distributions of the normalized vorticity magnitude $\sqrt{\omega_{i}\omega_{i}}\times h/u_{\tau ,NR}$ at z/h=4 are shown in the background. The signatures of wall ejections can be seen from the distributions of $\sqrt{\omega_{i}\omega_{i}}\times h/u_{\tau ,NR}$. The near-wall flow dynamics, in turn, affect the wrinkling of flame surface as it propagates towards the wall. In the case of isothermal wall boundary condition, the heat loss through the wall gives rise to quenching when the flame comes in the vicinity of the wall (e.g., $t/t_{f}=14.70$ where $t_{f}=\delta_{th}/S_{L}$ is the chemical timescale). In the case of adiabatic wall boundary condition, the flame eventually extinguishes when all the reactants are consumed. This can be seen from the broken islands of c=0.5 isosurface at $t/t_{f}=14.70$ even in the case of the adiabatic wall. It can further be seen by comparing different time instants in Figure 2 that the vorticity magnitude close to the wall decreases as the flame propagates towards the wall because of the decay in vorticity towards the burned gas side as a result of dilatation rate and viscous action [30,41]. It can be appreciated from Figure 2 that significant changes in thermodynamic state take place during head-on wall interaction of turbulent premixed flames irrespective of the thermal boundary condition at the wall.

**Figure 3:**
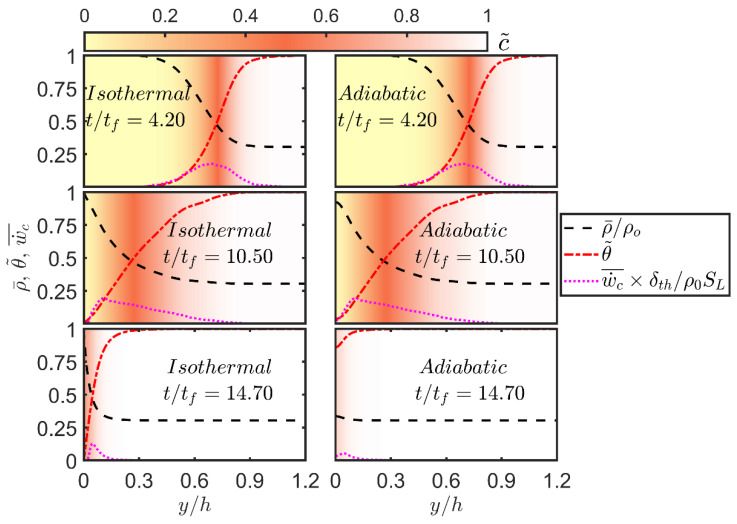
Variations of normalised mean density $\bar{\rho }/\rho_{0}$, Favre-averaged non-dimensional temperature $\tilde{\theta }=\left(\tilde{T}-T_{0}\right)/\left(T_{ad}-T_{0}\right)$, Reynolds averaged reaction rate $\bar{{\dot{w}}_{c}}\times \delta_{th}/\rho_{0}S_{L}$ with the normalised wall-normal distance y/h for isothermal (**left**) and adiabatic (**right**) wall boundary conditions at $t/t_{f}=4.20$, 10.50, 14.70 (**top** to **bottom**). The background is coloured by the values of $\tilde{c}$.

In order to demonstrate the aforementioned state changes, the variations of Reynolds averaged values of normalised density $\bar{\rho }/\rho_{0}$, Favre-averaged non-dimensional temperature $\tilde{\theta }=\left(\tilde{T}-T_{0}\right)/\left(T_{ad}-T_{0}\right)$ and Reynolds averaged reaction rate of reaction progress variable $\bar{{\dot{w}}_{c}}\times \delta_{th}/\rho_{0}S_{L}$ in the wall-normal direction are shown in Figure 3 for different time instants. The background in Figure 3 is coloured by the local values of $\tilde{c}$. It can be seen from Figure 3 that the equality between $\tilde{c}$ and $\tilde{\theta }$ holds when the flame remains away from the wall (e.g., $t/t_{f}=4.20$) but the coupling between $\tilde{c}$ and $\tilde{\theta }$ is lost during flame-wall interaction in the case of the isothermal boundary condition while $\tilde{c}=\tilde{\theta }$ is maintained at all stages in the case of the adiabatic boundary condition. In the case of the isothermal boundary condition, $\tilde{\theta }$ remains 0.0 at the wall but $\tilde{c}$ at the wall continues to increase during FWI even after flame quenching (i.e., even after $\bar{{\dot{w}}_{c}}$ disappears) because of diffusion of unburned reactants from the wall to the interior of the domain in the absence of chemical reaction. Figure 3 shows that $\bar{{\dot{w}}_{c}}$ vanishes in the isothermal case due to heat loss through the wall once the flame reaches in the vicinity of it. Moreover, $\bar{{\dot{w}}_{c}}$ remains vanishingly small at the wall at all stages in the isothermal case but $\bar{{\dot{w}}_{c}}$ assumes non-zero values at the wall during head-on interaction in the adiabatic case which is also accompanied by the increases in $\tilde{c}$ and $\tilde{\theta }$ until the fuel is fully consumed, which is followed by a decrease in $\bar{{\dot{w}}_{c}}$ at the wall. The density drops with an increase in $\tilde{\theta }$ and in the isothermal case $\bar{\rho }=\rho_{0}$ is maintained at the wall, whereas $\bar{\rho }$ decreases at the wall and remains smaller than the unburned gas density $\rho_{0}$ with the progress of head-on interaction in the case of the adiabatic wall. The increase in temperature and decrease in density for a large portion for the flow domain in this configuration suggests that the extensive entropy of the domain increases with the progress of head-on interaction for both isothermal and adiabatic cases but the differences in $\tilde{\theta }$ and $\bar{\rho }$ at the wall between these cases suggest that the entropy changes of the gases will be different depending on the thermal boundary condition.

**Figure 4:**
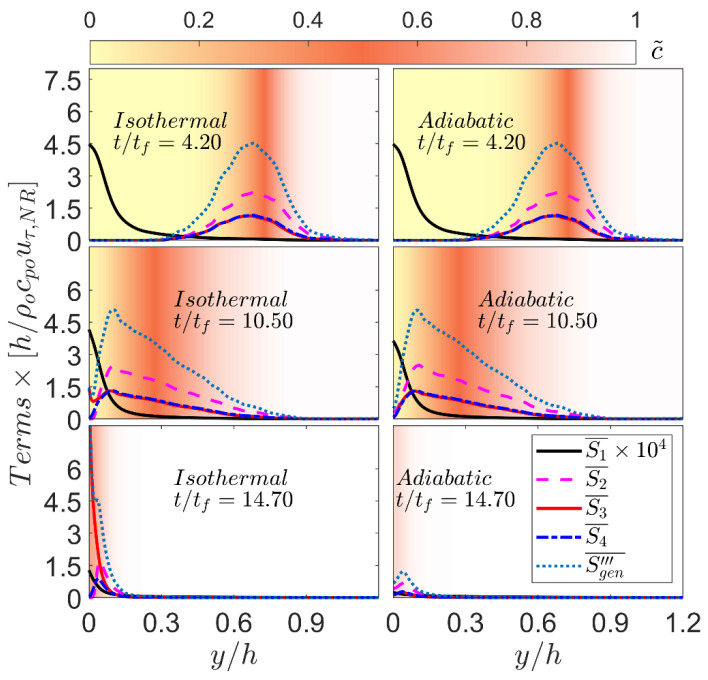
Variations of Reynolds averaged normalised values of different entropy generation contributions (i.e., $\left\{{\bar{S}}_{1},{\bar{S}}_{2},{\bar{S}}_{3},{\bar{S}}_{4},\bar{S_{gen}^{\prime \prime \prime }}=\left({\bar{S}}_{1}+{\bar{S}}_{2}+{\bar{S}}_{3}+{\bar{S}}_{4}\right)\right\}\times h/\rho_{0}c_{p0}u_{\tau ,NR}$) with the normalised wall-normal distance y/h for isothermal (**left**) and adiabatic (**right**) wall boundary conditions at $t/t_{f}=4.20$, 10.50, 14.70 (**top** to **bottom**). The background is coloured by the values of $\tilde{c}$.

The variations of the Reynolds averaged normalized values of different entropy generation contributions (i.e., $\left\{{\bar{S}}_{1},{\bar{S}}_{2},{\bar{S}}_{3},{\bar{S}}_{4},\bar{S_{gen}^{\prime \prime \prime }}=\left({\bar{S}}_{1}+{\bar{S}}_{2}+{\bar{S}}_{3}+{\bar{S}}_{4}\right)\right\}\times h/\rho_{0}c_{p0}u_{\tau ,NR}$) in the normalised wall normal direction y/h at different time instants for both isothermal and adiabatic boundary conditions are shown in Figure 4. It can be seen from Figure 4 that the magnitude of the entropy generation due to viscous action ${\bar{S}}_{1}$ assumes the highest magnitude at the wall but its magnitude remains negligible in comparison to the magnitudes of the entropy generation contributions due to thermal diffusion and molecular mixing (i.e., ${\bar{S}}_{3}$ and ${\bar{S}}_{4}$) at all stages of head-on interaction irrespective of the wall boundary condition. The values of ${\bar{S}}_{3}$ and ${\bar{S}}_{4}$ remain comparable to that of ${\bar{S}}_{2}$ when the flame is away from the wall (e.g., $t/t_{f}=4.20$) for both boundary conditions and the contribution of entropy generation due to chemical reaction ${\bar{S}}_{2}$ acts as a leading entropy generation mechanism at this time (e.g., $t/t_{f}=4.20$) for both isothermal and adiabatic cases. The variations of ${\bar{S}}_{1},{\bar{S}}_{2},{\bar{S}}_{3},{\bar{S}}_{4},\bar{S_{gen}^{\prime \prime \prime }}$ at $t/t_{f}=4.20$, when the flame remains away from the wall are found to be similar to the corresponding variations in a 1D unstretched globally adiabatic laminar flame (not shown here). Moreover, the variations of ${\bar{S}}_{1},{\bar{S}}_{2},{\bar{S}}_{3},{\bar{S}}_{4},\bar{S_{gen}^{\prime \prime \prime }}$, when the flame remains away from the wall, remain qualitatively similar to the previous findings in turbulent premixed flames without walls [18].

In the case of isothermal boundary condition, ${\bar{S}}_{3}$ and ${\bar{S}}_{4}$ assume similar values when the flame is away from wall (i.e., $t/t_{f}=4.20$), but ${\bar{S}}_{3}$ assumes greater values than ${\bar{S}}_{4}$ in the vicinity of the wall (i.e., $t/t_{f}=10.50$) and this trend strengthens with the progress in time (e.g., compare between $t/t_{f}=10.50$ and 14.70). By contrast, ${\bar{S}}_{3}$ and ${\bar{S}}_{4}$ remain close to each other and show the same qualitative behaviour for the adiabatic wall boundary condition.

Scaling the fluctuating parts of $\tau_{ij}$ and $\partial u_{i}/\partial x_{j}$ by $\rho_{0}S_{L}\delta_{th}u^{\prime }/\Lambda$ and $u^{\prime }/\Lambda$ according to Tennekes and Lumley [42] (where Λ is the Taylor Microscale ) leads to: ${\bar{S}}_{1}\sim \rho_{0}c_{p0}S_{L}/\delta_{th}\times \left(\gamma -1\right)\times Ma_{S}^{2}\left(\tilde{\varepsilon }\delta_{th}/S_{L}^{3}\right)\sim \rho_{0}c_{p0}S_{L}/\delta_{th}\times \left(\gamma -1\right)\times Ma_{S}^{2}Ka^{2}$ where γ is the ratio of specific heats, $Ma_{S}=S_{L}/\sqrt{\gamma RT_{0}}$ is a Mach number based on laminar burning velocity $S_{L}$ and unburned gas temperature $T_{0}$, and $\tilde{\varepsilon }=\bar{\mu \left(\partial u_{i}^{\prime \prime }/\partial x_{j}\right)\left(\partial u_{i}^{\prime \prime }/\partial x_{j}\right)}/\bar{\rho }$ is the dissipation rate of turbulent kinetic energy $\tilde{k}=\bar{\rho u_{i}^{\prime \prime }u_{i}^{\prime \prime }}/2\bar{\rho }$. Scaling scalar gradients with respect to $\delta_{th}$ yields: ${\bar{S}}_{2}\sim \rho_{0}c_{p0}S_{L}/\delta_{th}$; ${\bar{S}}_{3}\sim \rho_{0}c_{p0}S_{L}/\delta_{th}$ and ${\bar{S}}_{4}\sim \rho_{0}c_{p0}S_{L}/\delta_{th}$ [18,19]. These scalings suggest that ${\bar{S}}_{1}$ is expected to be much smaller than ${\bar{S}}_{2}$, ${\bar{S}}_{3}$ and ${\bar{S}}_{4}$ within the flame because Ka<1 and $Ma_{S}\ll 1$ in the flames considered here. Moreover, these scalings suggest that the magnitudes of ${\bar{S}}_{2}$, ${\bar{S}}_{3}$ and ${\bar{S}}_{4}$ are expected to be comparable within the flame, which is consistent with the findings at $t/t_{f}=4.20$ when the flame is away from the wall. However, ${\bar{S}}_{2}$ eventually disappears due to flame quenching in the isothermal case (e.g., $t/t_{f}=14.70$) where ${\bar{S}}_{3}$ becomes the most significant contributor to $\bar{S_{gen}^{\prime \prime \prime }}$ and assumes the highest magnitude at the wall. The magnitudes of ${\bar{S}}_{3}$ and ${\bar{S}}_{4}$ remain comparable away from the wall even after flame quenching. By contrast, the magnitudes of ${\bar{S}}_{3}$ and ${\bar{S}}_{4}$ remain comparable and their peak values are obtained away from the wall at every stage of head-on interaction in the adiabatic case. Accordingly, $\bar{S_{gen}^{\prime \prime \prime }}$ assumes a peak value away from the wall in the case of adiabatic boundary condition, whereas $\bar{S_{gen}^{\prime \prime \prime }}$ peaks at the wall during flame quenching (e.g., $t/t_{f}=14.70$) in the isothermal wall case.

**Figure 5:**
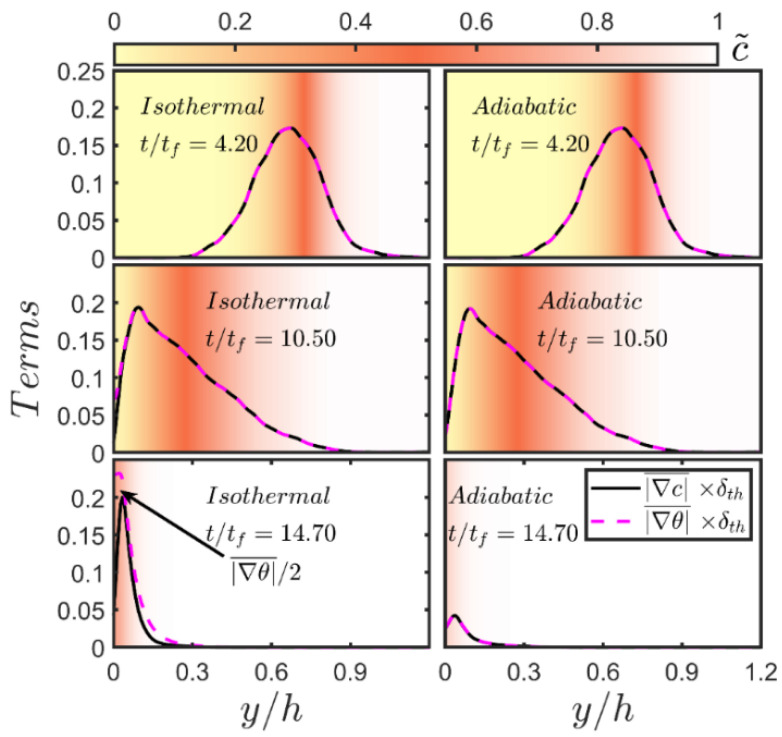
Variations of $\bar{\left|\nabla c\right|}\times \delta_{th}$ and $\bar{\left|\nabla \theta \right|}\times \delta_{th}$ with the normalised wall-normal distance y/h for isothermal (**left**) and adiabatic (**right**) wall boundary conditions at $t/t_{f}=4.20$, 10.50, 14.70 (**top** to **bottom**). The background is coloured by the values of $\tilde{c}$. The non-dimensional temperature gradient $\bar{\left|\nabla \theta \right|}$ in the isothermal case is divided by 2.0 to bring it to the same scale as $\bar{\left|\nabla c\right|}$ at $t/t_{f}=14.70$.

In order to explain the differences in the behaviour of ${\bar{S}}_{3}$ and ${\bar{S}}_{4}$ between the isothermal and adiabatic conditions, the wall-normal distributions of $\bar{\left|\nabla c\right|}\times \delta_{th}$ and $\bar{\left|\nabla \theta \right|}\times \delta_{th}$ at different time instants are shown for both wall boundary conditions in Figure 5 because ${\bar{S}}_{3}$ and ${\bar{S}}_{4}$ can be scaled using $\rho_{0}c_{p0}S_{L}\bar{\left|\nabla \theta \right|}$ and $\rho_{0}c_{p0}S_{L}\bar{\left|\nabla c\right|}$, respectively [18]. It can be seen from Figure 5 that $\bar{\left|\nabla c\right|}\times \delta_{th}$ and $\bar{\left|\nabla \theta \right|}\times \delta_{th}$ assume identical values away from the wall in both wall boundary conditions. For low Mach number, unity Lewis number conditions, c and θ remain almost equal to each other where the effects of wall heat loss are not significant and therefore $\bar{\left|\nabla c\right|}\times \delta_{th}$ and $\bar{\left|\nabla \theta \right|}\times \delta_{th}$ remain almost equal to each other when the flame is away from the wall in the case of isothermal wall and $\bar{\left|\nabla c\right|}\approx \bar{\left|\nabla \theta \right|}$ is maintained at all stages of head-on interaction in the case of adiabatic boundary condition.

**Figure 6:**
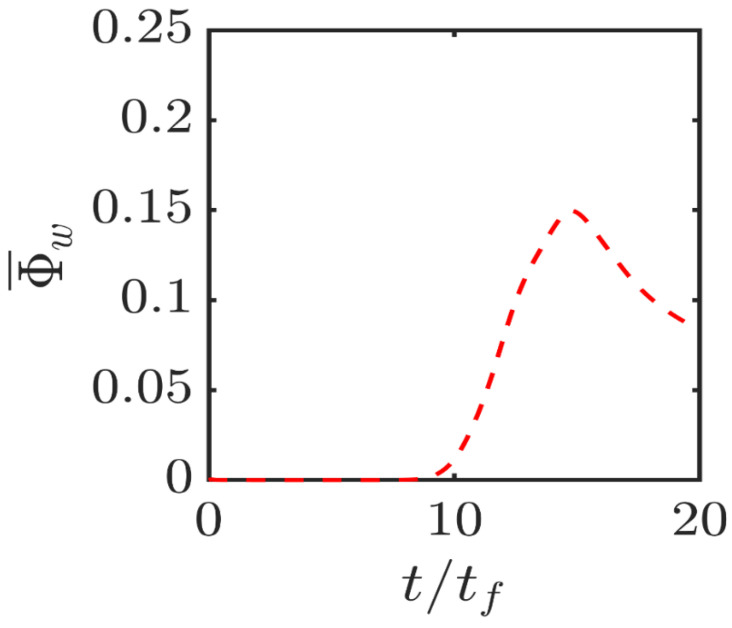
Variations of the normalised wall heat flux magnitude ${\bar{\Phi }}_{w}=q_{w}/\left[\rho_{0}c_{p0}u_{\tau ,NR}\left(T_{ad}-T_{0}\right)\right]$ with time for the isothermal wall boundary condition.

The temporal evolution of the normalised wall heat flux magnitude ${\bar{\Phi }}_{w}=q_{w}/\left[\rho_{0}c_{p0}u_{\tau ,NR}\left(T_{ad}-T_{0}\right)\right]$ for the isothermal wall case is shown in Figure 6. A comparison between Figure 5 and Figure 6 reveals that $\bar{\left|\nabla c\right|}\approx \bar{\left|\nabla \theta \right|}$ is maintained when the flame is away from the wall and ${\bar{\Phi }}_{w}$ assumes negligible values but this equality is lost when ${\bar{\Phi }}_{w}$ takes significant values indicating heat loss through the wall, which prompts a drop in $\bar{{\dot{w}}_{c}}$ in the near-wall region as a result of flame quenching (see Figure 3). This is also consistent with the loss of equality between $\tilde{c}$ and $\tilde{\theta }$ during head-on interaction in the case of isothermal wall (see Figure 3). During flame quenching in the case of isothermal wall $\bar{\left|\nabla \theta \right|}$ assumes greater values than $\bar{\left|\nabla c\right|}$ close to the wall due to high temperature gradient at the wall because of flame quenching (note the quenching distance $\delta_{Q}$ is about $0.8\delta_{th}$ in this case), whereas the wall-normal component of ∇c vanishes due to impenetrability, which leads to $\bar{\left|\nabla \theta \right|}>\bar{\left|\nabla c\right|}$ in the isothermal wall boundary condition. By contrast, wall normal components of ∇θ and ∇c vanish at the wall in the case of adiabatic wall boundary condition, which gives rise to comparable values of $\bar{\left|\nabla \theta \right|}$ and $\bar{\left|\nabla c\right|}$ at the wall and accordingly comparable magnitudes of ${\bar{S}}_{3}$ and ${\bar{S}}_{4}$ are obtained close to the wall in the case of the adiabatic wall boundary condition.

It can further be seen from Figure 5 that $\bar{\left|\nabla c\right|}$ drops at the wall during the head-on interaction for both wall boundary conditions. However, the peak values of $\bar{\left|\nabla \theta \right|}$ and $\bar{\left|\nabla c\right|}$ at the advanced stages of head-on interaction (e.g., $t/t_{f}=14.70$) remain greater in the case of isothermal wall boundary condition than in the case of an adiabatic wall. The flame quenching in the case of isothermal wall sets up steeper gradients of c and θ close to the wall than those in the case of adiabatic wall boundary condition. The physical mechanisms responsible for the reduction in |∇c| at the wall in both isothermal and adiabatic boundary conditions are explained elsewhere [29], which are not repeated here. Interested readers are referred to Ahmed et al. [29] for further information in this regard. The reduction in |∇c| during head-on interaction of the flame gives rise to the reduction in the magnitude of ${\bar{S}}_{4}\sim \rho_{0}c_{p0}S_{L}\bar{\left|\nabla c\right|}$ at the wall in comparison to the values, which are obtained when the flame remains away from the wall.

**Figure 7:**
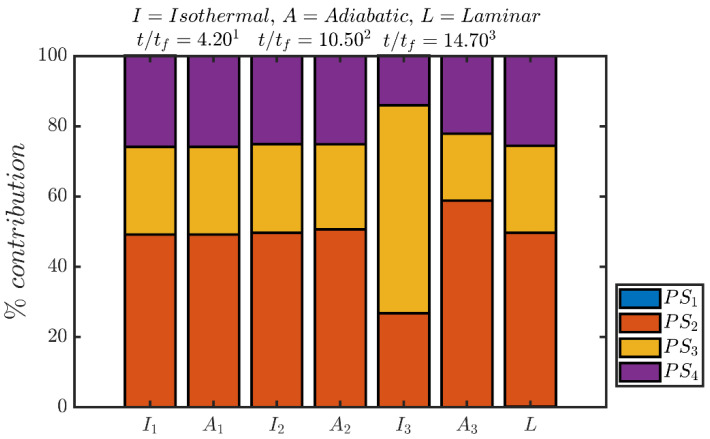
Variations of percentage shares of different entropy generation mechanisms (i.e., $PS_{i}=\int_{V_{f}}S_{i}dV/\int_{V_{f}}S_{gen}^{\prime \prime \prime }dV\times 100\%$ for i=1, 2, 3, 4) to the overall entropy generation rate $\int_{V_{f}}S_{gen}^{\prime \prime \prime }dV=\int_{V_{f}}\left(S_{1}+S_{2}+S_{3}+S_{4}\right)dV$ within the flame brush at $t/t_{f}=4.20$, 10.50, 14.70 (from **left** to **right**) where the first (second) bar of each pair represents the isothermal (adiabatic) boundary conditions. The corresponding percentage shares for a 1D unstretched globally adiabatic laminar premixed flame are also shown in the above figures (far **right**) for the sake of comparison.

The percentage shares of different entropy generation mechanisms (i.e., $PS_{i}=\int_{V_{f}}S_{i}dV/\int_{V_{f}}S_{gen}^{\prime \prime \prime }dV\times 100\%$ for i=1, 2, 3, 4) to the overall entropy generation rate $\int_{V_{f}}S_{gen}^{\prime \prime \prime }dV$ within the flame brush at different time instants are shown in Figure 7 for both isothermal and adiabatic wall boundary conditions. Figure 7 shows that the percentage share of viscous action (i.e., $\int_{V_{f}}S_{1}dV$) remains negligible at all times for both cases and the percentage shares by chemical reaction (i.e., $\int_{V_{f}}S_{2}dV$), thermal diffusion (i.e., $\int_{V_{f}}S_{3}dV$) and molecular mixing (i.e., $\int_{V_{f}}S_{4}dV$) remain comparable for the case with adiabatic wall, which is consistent with previous findings for turbulent premixed flames without walls [18]. For the isothermal wall boundary condition comparable percentage contributions of chemical reaction (i.e., $\int_{V_{f}}S_{2}dV$), thermal diffusion (i.e., $\int_{V_{f}}S_{3}dV$) and molecular mixing (i.e., $\int_{V_{f}}S_{4}dV$) are obtained when the flame is away from the wall. However, the percentage share of $\int_{V_{f}}S_{2}dV$ drops when the flame starts to quench (e.g., $t/t_{f}=14.70$) and thermal diffusion (i.e., $\int_{V_{f}}S_{3}dV$) contribution becomes the major contributor to the entropy generation although molecular mixing contribution (i.e., $\int_{V_{f}}S_{4}dV$) continues to play a significant role.

In the case of adiabatic boundary condition, the percentage share of $\int_{V_{f}}S_{2}dV$ to the overall entropy generation increases, whereas the percentage shares of $\int_{V_{f}}S_{3}dV$ and $\int_{V_{f}}S_{4}dV$ decrease when the flame interacts with the wall (e.g., $t/t_{f}=14.70$). This is a consequence of small values of |∇θ| and |∇c| during FWI (e.g., $t/t_{f}=14.70$) in the case of adiabatic boundary condition, which leads to small values of ${\bar{S}}_{3}$ and ${\bar{S}}_{4}$ (see Figure 4 and Figure 5). Accordingly the percentage share of $\int_{V_{f}}S_{3}dV$ and $\int_{V_{f}}S_{4}dV$ decreases and the percentage share of $\int_{V_{f}}S_{2}dV$ rises at $t/t_{f}=14.70$ in comparison to the conditions, which prevail at earlier time instants (e.g., $t/t_{f}=4.20$ and 10.50) when the flame remains away from the wall. These percentage shares of different entropy generation mechanisms (i.e., $\int_{V_{f}}S_{i}dV$ for i=1,2,3,4) are consistent with the variations of ${\bar{S}}_{i}$ in Figure 4, as $\int_{V_{f}}S_{i}dV$ can alternatively expressed as: $\int_{V_{f}}S_{i}dV=\int_{V_{f}}{\bar{S}}_{i}dV$ for i=1, 2, 3, 4.

**Figure 8:**
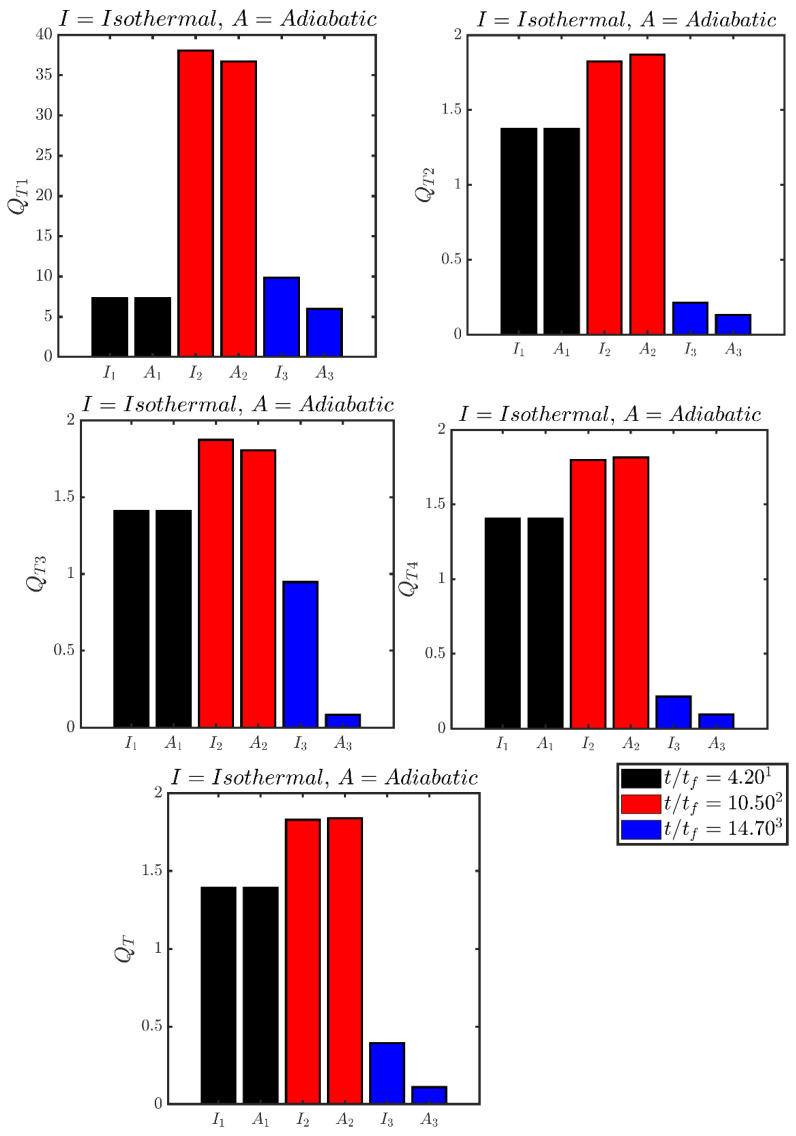
Variations of the augmentations of entropy generation in comparison to the unstretched laminar flame $Q_{T1},Q_{T2},Q_{T3},Q_{T4}$ and $Q_{T}$ at $t/t_{f}=4.20$, 10.50, 14.70 (from **left** to **right**) where the first (second) bar of each pair represents the isothermal (adiabatic) boundary conditions.

The variations of the augmentations of entropy generation in comparison to the unstretched laminar flame $Q_{T1},Q_{T2},Q_{T3},Q_{T4}$ and $Q_{T}$ at different time instants are presented in Figure 8, which shows that the entropy generation enhancement under turbulence is the highest for the viscous action $Q_{T1}$. The values of $Q_{T2},Q_{T3}$ and $Q_{T4}$ remain comparable when the flame is away from the wall (e.g., $t/t_{f}=4.20$) for both boundary conditions. They increase at $t/t_{f}=10.50$ when the flame propagates towards the wall but does not yet get significantly affected by it. This is a consequence of an increase in the turbulent flame brush thickness because of increased flame wrinkling induced by the near wall vortical motion. The values of $Q_{T2}$ and $Q_{T4}$ drop with time and assume values smaller than unity as the flame comes close to the wall and starts to interact with it, which is in accordance with the behaviours of ${\bar{S}}_{2}$ and ${\bar{S}}_{3}$ as shown in Figure 4. This trend is particularly strong for the adiabatic case where the reaction rate drops with time due to consumption of reactants which also leads to a reduction of $Q_{T2}$ with the progress of head-on interaction. By contrast, $Q_{T2}$ drops due to flame quenching in the case of isothermal wall boundary condition. The disappearances of wall-normal gradients of temperature, species mass and mole fractions contribute to smaller values of $Q_{T3}$ and $Q_{T4}$ in the adiabatic case in comparison to those in the isothermal case. The high temperature gradient and high magnitude of ${\bar{S}}_{3}$ at the wall due to flame quenching in the isothermal case (see Figure 4 and Figure 5) contribute to higher values of $Q_{T3}$ than in the adiabatic case. It is also worthwhile to note that the magnitude of $Q_{T}$ is comparable to those of $Q_{T2},Q_{T3},Q_{T4}$ although $Q_{T1}$ is much greater than $Q_{T2},Q_{T3},Q_{T4}$. This behaviour is an outcome of the negligible contribution of ${\bar{S}}_{1}$ towards $\bar{S_{gen}^{\prime \prime \prime }}$ (see Figure 4). It can be seen from Figure 8 that $Q_{T}$ remains smaller in the adiabatic case than in the case of isothermal wall when the flame interacts with the wall (e.g., $t/t_{f}=14.70$), which is consistent with higher values of $\bar{S_{gen}^{\prime \prime \prime }}$ in the isothermal case at $t/t_{f}=14.70$. Thus, the overall thermodynamic irreversibility generation is smaller in the case of adiabatic walls than in the isothermal case during the head-on FWI.

**Figure 9:**
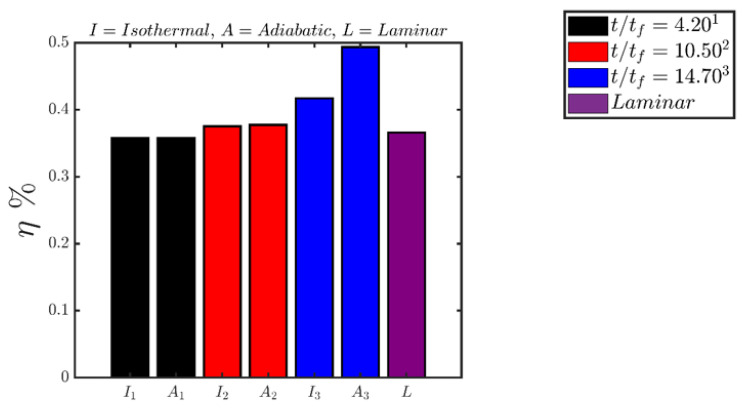
Variations of the second-law efficiency $\eta_{II}$ at $t/t_{f}=4.20$, 10.50, 14.70 (from **left** to **right**) where the first (second) bar of each pair represents the isothermal (adiabatic) boundary conditions. The second-law efficiency $\eta_{II}$ for a 1D unstretched globally adiabatic laminar premixed flame is also shown (far **right**) in the above figures for the sake of comparison.

Finally, the evolutions of the second-law efficiency $\eta_{II}$ with the progress of head-on interaction for both boundary conditions are shown in Figure 9. It can be seen from Figure 9 that $\eta_{II}$ values for both isothermal and adiabatic conditions remain comparable when the flame is away from the wall (e.g., $t/t_{f}=4.20$). Moreover, the values of $\eta_{II}$, when the flame is away from the wall, remain comparable to that of the unstretched laminar premixed flame under globally adiabatic condition. The second-law efficiency $\eta_{II}$ marginally increases at $t/t_{f}=10.50$ when the flame propagates towards the wall but does not get affected by it. This is a consequence of an increase in the overall burning rate as a result of increased flame wrinkling, which increases the magnitude of $\int_{V_{f}}\sum_{i=1}^{n}\left(-a_{\alpha }{\dot{w}}_{\alpha }\right)dV$ in comparison to $\int_{V_{f}}T_{0}S_{gen}^{\prime \prime \prime }dV$. The second law efficiency increases with time as the head-on interaction with the wall (e.g., $t/t_{f}=14.70$) progresses with time because of reduced magnitudes of entropy generation rates due to chemical reaction (see Figure 4 and Figure 7) in comparison to $\int_{V_{f}}\sum_{i=1}^{n}\left(-a_{\alpha }{\dot{w}}_{\alpha }\right)dV$.

The findings from Figure 4, Figure 7, Figure 8 and Figure 9 suggest that the thermal boundary condition at the wall has a major impact on the entropy generation rate and its augmentation in comparison to the corresponding values in globally adiabatic unstretched laminar premixed flame without walls. Thus, the thermal wall boundary condition can significantly affect the entropy generation of the combustor and, thus, there is a scope for the optimization of wall cooling from the point of view of ensuring structural integrity and minimization of thermodynamic irreversibility in practical applications.

## 5. Conclusions

The statistical behaviours of different entropy generation mechanisms due to viscous action, chemical reaction, thermal diffusion and molecular mixing in the case of head-on interaction of turbulent premixed flames with a chemically inert wall in a turbulent boundary layer corresponding to $Re_{\tau }=110$ have been analysed using three-dimensional DNS data. The entropy generation characteristics and the second-law efficiency in this configuration have been analysed for both isothermal and adiabatic thermal boundary conditions for the wall. The main conclusions of this analysis are listed as follows:

- It has been found that the contribution of the viscous action to the overall entropy generation rate remains negligible at all stages of head-on interaction for both isothermal and adiabatic boundary conditions.
- The simulation results reveal that the mean entropy generation rates by chemical reaction, thermal diffusion and species diffusion remain comparable within the flame when it remains sufficiently away from the wall. This is found to be consistent with previous findings for turbulent premixed flames without walls [18].
- The percentage shares of entropy generation rates by viscous action, chemical reaction, thermal diffusion and molecular mixing in turbulent premixed flames away from the wall remain also comparable to that in the unstretched laminar premixed flame under globally adiabatic conditions. However, the entropy generation due chemical reaction decreases during FWI for both isothermal and adiabatic boundary conditions. The reaction rate drops at the wall due to consumption of the reactants for adiabatic boundary condition, which leads to a reduction of the entropy generation rate due to chemical reaction during FWI. By contrast, the reaction rate vanishes at the wall in the isothermal case due to flame quenching, which also leads to a reduction in entropy generation due to chemical reaction.
- The entropy generation due to thermal diffusion during advanced stages of FWI remains relatively stronger in the case of isothermal wall boundary condition than in the adiabatic case due to the high temperature gradient induced by flame quenching in the isothermal wall case. By contrast, the mean values of entropy generation due to thermal diffusion and molecular mixing remain small close to the wall even when the flame interacts with the wall under adiabatic boundary condition. This behaviour arises because the wall-normal components of the gradients of temperature and species vanish at the wall.
- The differences in entropy generation in response to thermal wall boundary conditions affect the overall thermodynamic irreversibility generation and second-law efficiency of the FWI process in turbulent boundary layers.
- The second-law efficiency has been found to increase during advanced stages of FWI because of the reduced entropy generation due to chemical reaction and molecular mixing.
- It is worth noting that the present analysis has been conducted for simple chemistry and transport as this is the first study which analysed entropy generation statistics during FWI within turbulent boundary layers. This study revealed that the entropy generation statistics is significantly affected by flame quenching in the case of isothermal walls. In addition, this study reveals for the first time that the wall boundary condition, significantly affects the entropy generation rate in FWI principally due to differences in thermal diffusion and chemical reaction contributions to entropy generation at the wall and its vicinity.
- The present findings indicate that the thermal condition prevailing at the combustor wall can significantly affect the thermodynamic performance of the combustor and, thus, the wall cooling needs to be optimized from the point of view of ensuring structural integrity and minimization of thermodynamic irreversibility in practical applications.

Although it was demonstrated in previous analyses [32,33] that the flame quenching distance, wall heat flux, scalar gradient and mean reaction rate statistics in flame-wall interaction for multi-step skeletal chemical mechanism remain qualitatively similar to that of simple chemistry and transport, further analyses with detailed chemistry and transport will be needed for quantitative predictions of entropy generation rate and second law efficiency. This will form the basis of future investigations.

## Data Availability

The data can be obtained from the authors based on reasonable requests.
